# High-Pressure Orthorhombic Ferromagnesite as a Potential Deep-Mantle Carbon Carrier

**DOI:** 10.1038/srep07640

**Published:** 2015-01-06

**Authors:** Jin Liu, Jung-Fu Lin, Vitali B. Prakapenka

**Affiliations:** 1 Department of Geological Sciences, Jackson School of Geosciences, The University of Texas at Austin, Austin, TX 78712, USA; 2 Center for High Pressure Science and Technology Advanced Research (HPSTAR), Shanghai 201203, People's Republic of China; 3 Consortium for Advanced Radiation Sources, The University of Chicago, Chicago, IL 60637, USA

## Abstract

Knowledge of the physical and chemical properties of candidate deep-carbon carriers such as ferromagnesite [(Mg,Fe)CO_3_] at high pressure and temperature of the deep mantle is necessary for our understanding of deep-carbon storage as well as the global carbon cycle of the planet. Previous studies have reported very different scenarios for the (Mg,Fe)CO_3_ system at deep-mantle conditions including the chemical dissociation to (Mg,Fe)O+CO_2_, the occurrence of the tetrahedrally-coordinated carbonates based on CO_4_ structural units, and various high-pressure phase transitions. Here we have studied the phase stability and compressional behavior of (Mg,Fe)CO_3_ carbonates up to relevant lower-mantle conditions of approximately 120 GPa and 2400 K. Our experimental results show that the rhombohedral siderite (Phase I) transforms to an orthorhombic phase (Phase II with *Pmm*2 space group) at approximately 50 GPa and 1400 K. The structural transition is likely driven by the spin transition of iron accompanied by a volume collapse in the Fe-rich (Mg,Fe)CO_3_ phases; the spin transition stabilizes the high-pressure phase II at much lower pressure conditions than its Mg-rich counterpart. It is conceivable that the low-spin ferromagnesite phase II becomes a major deep-carbon carrier at the deeper parts of the lower mantle below 1900 km in depth.

The existence of carbon-bearing solids, fluids, and melts in the Earth's deep interior can affect a series of geophysical and geochemical properties of our planet^1^,^2^,^3^,^4^,^5^. Laboratory studies of carbon-bearing minerals at high pressures and temperatures (*P-T*) can thus provide crucial constraints on the role and behavior of the deep-carbon phases in the Earth's mantle as well as the mantle's role in the global carbon cycle^6^,^7^. Among all carbonates that can be potentially subducted into the Earth's deep mantle through plate subductions, iron-bearing magnesite [(Mg,Fe)CO_3_] has been proposed to be a major deep-carbon host because of its existence in subducting plates as well as its wide *P-T* stability field^8^,^9^,^10^,^11^,^12^. (Mg,Fe)CO_3_ forms a continuous solid solution between magnesite [MgCO_3_] and siderite [FeCO_3_] in the rhombohedral *R*
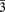
*c* structure, in which the Mg-rich compositions are called ferromagnesite and the Fe-rich part is named magnesiosiderite.

A number of previous studies have reported different behaviors of the (Mg,Fe)CO_3_ system at relevant *P-T* conditions of the mantle^10^,^13^,^14^,^15^,^16^,^17^,^18^; various high-pressure (Mg,Fe)CO_3_ polymorphic phase transitions have been reported to occur at high *P-T* including the formation of phases based on tetrahedral CO_4_ units (similar to the SiO_4_ units of silicate minerals)^14^,^15^, and the chemical decomposition of (Mg,Fe)CO_3_ into (Mg,Fe)O and CO_2_ (refs. 9,18). It has been experimentally observed that the rhombohedral structure of the end-member magnesite is stable up to approximately 100 GPa and 2000 K, but then transforms into an orthorhombic structure, named magnesite II, at approximately 115 GPa and 2100 K (ref. 10). Theoretical calculations^13^,^14^,^15^ and other experimental studies^16^, on the other hand, have shown that magnesite transforms into a high-pressure phase built with (CO_4_)^4−^ tetrahedral groups energetically favored in the monoclinic structure. At high *P-T* conditions, (Mg,Fe)CO_3_ and a mixture of oxides ((Mg,Fe)O and CO_2_) have been suggested to transform into a number of Fe^3+^-bearing high-pressure carbonate, magnetite, and nano-diamonds via intra-crystalline reactions between Fe^2+^ and (CO_3_)^2–^ (ref. 16). A new high *P-T* phase with the Fe_4_(CO_4_)_3_ chemical formula and all iron as Fe^3+^ was reported to occur through high-pressure reactions between FeO and saturated CO_2_ at approximately 40 GPa and 1500 K (ref. 17). After temperature quenching to room temperature, this new phase undergoes a structural transformation in compression^17^. These experimental results suggest that the oxygen fugacity in the (Mg,Fe)CO_3_ system may strongly affect the reaction products at high *P-T* conditions^18^,^19^. In the Earth's mantle, the oxygen fugacity (*f*O_2_) may be controlled by the carbon-oxygen-hydrogen-sulfur and ferric-ferrous iron equilibria, resulting in a general decrease in *f*O_2_ with depth through the reduction of volatile species, such that the ferric iron content can be used as an indicator for the redox state of the Earth's interior (see ref. 20 for a review). However, the self-disproportionation model of Fe^2+^ into Fe^3+^ and Fe^0^ has been presented as a mechanism acting in the lower mantle where the formation of Al-bearing silicate perovskite is associated with the disproportionation of Fe^2+^ into Fe^3+^ in perovskite and metallic Fe (a charge balanced reaction). Thus far, the exact crystal structure of the high-pressure phase in the (Mg,Fe)CO_3_ system and the C-O coordination geometry remain highly debated^10^,^13^,^14^,^15^,^16^. Since the lower-mantle magnesite is expected to contain a certain amount of siderite in the solid solution^21^, studying the phase stability, crystal structure, and thermal equation of state (EoS) of ferromagnesite, especially through the recently discovered spin transition of iron^22^,^23^,^24^, at relevant *P-T* conditions of the deep mantle is of particular importance to our understanding of the deep-carbon storage^6^,^25^.

An iso-symmetric electronic spin transition of iron in rhombohedral (Mg,Fe)CO_3_ has been reported to occur at approximately 45 GPa and high temperatures^11^,^12^,^26^,^27^. Evaluation of the pressure-volume relationship of siderite across the spin transition showed that the spin transition is associated with a volume collapse of approximately 10%, such that the low-spin siderite exhibits a smaller unit cell volume than the endmember MgCO_3 _counterpart^11^,^28^. That is, the low-spin siderite has an effective ionic radius of the Fe^2+^ smaller than that of the Mg^2+^ and is much denser than the rhombohedral MgCO_3_ and the extrapolated high-spin siderite^11^,^29^,^30^. Since the iron ions occupy the isolated octahedral sites in the rhombohedral lattice of siderite, the Fe^2+^-Fe^2+^ exchange interactions between neighboring Fe^2+^ likely have a negligible effect on the transition pressure from the high-spin state to the low-spin state^12^. The weak iron-iron exchange interactions as well as drastic reduction of the effective Fe^2+^ ionic radius in rhombohedral (Mg,Fe)CO_3_ raise the possibility that the low-spin siderite likely undergoes a structural transition at a relatively lower pressure than that of magnesite^11^,^30^. To decipher the phase stability of the (Mg,Fe)CO_3_ system in the lower mantle, here we have investigated the crystal structures of siderite and magnesiosiderite [(Fe_0.65_Mg_0.35_)CO_3_] samples at high *P-T* conditions using synchrotron X-ray diffraction (XRD) in a laser-heated diamond anvil cell (DAC). Together with electron microprobe analyses of the quenched samples, our results show a structural phase transition from the rhombohedral (space group: *R*
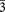
*c*) phase (Phase I) to the orthorhombic phase (Phase II) at above approximately 50 GPa and 1400 K. The *P-T* range for the structural transition in the Fe-rich parts of the (Mg,Fe)CO_3_ system can be understood in terms of the volume collapse due to the spin transition of iron at high *P-T*. Here we apply these results to decipher physical and chemical behaviors of candidate deep-mantle carbonates in the lower mantle.

## Experimental Results and Discussions

### High-Pressure Phase Transition in Siderite

**Figure 1:**
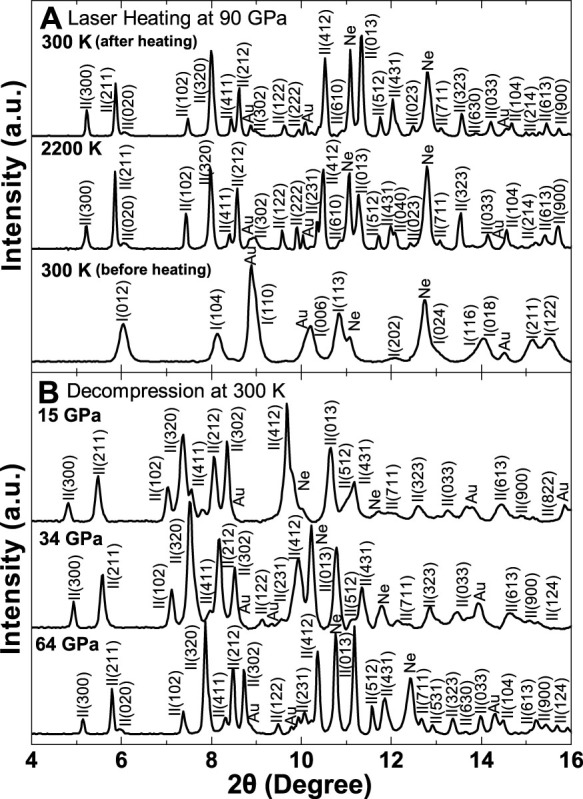
Representative X-ray diffraction patterns of siderite I and II phases [FeCO_3_] at high *P-T*. (*A*) FeCO_3_ heated up to 2200 K at 90 GPa. (*B*) Decompression of siderite II at room temperature. Gold (Au) was used as the primary pressure calibrant, while neon (Ne) was used as the thermal insulator, pressure medium as well as the secondary pressure calibrant^49^. Miller indices (hkl) of siderite I and II phases are labeled as I(hkl) and II(hkl), respectively. The wavelength of the monochromatic X-ray beam was 0.3344 Å.

**Figure 2:**
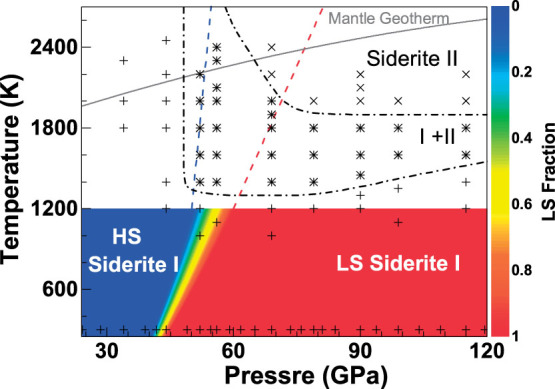
Schematic phase relations of siderite [FeCO_3_] at high *P-T* relevant to the lower mantle conditions. Experimental observations of the phases were collected from three different samples in this study, and are shown as black symbols (+, * and ×) (Table S1). Pluses (+): siderite I; plus/crosses (*): two-phase co-existing region (siderite I and II); crosses (×): siderite II; dash-dotted curves: transition boundaries between phase I and mixed I+II phases or phase II and mixed I+II phases. Spin crossover phase diagram of iron in siderite I below 1200 K from Liu et al.^27^ is plotted in colored area for comparison with the observations of the structural phases in this study. HS: high-spin state; LS: low-spin state. The color bar at the right represents the fraction of the LS state^27^. Dashed curves: the LS fraction of iron in siderite I extrapolated from 1200 K to 2700 K (blue: the initial occurrence of the LS state; red: the last occurrence of the HS state, ref. 27). Grey solid curve: expected lower-mantle geotherm^41^. The observation of the mixed-phase region may be due to *P-T* uncertainty as well as the sluggishness (the kinetic barrier) of the phase transformation.

XRD patterns were collected up to 120 GPa and 2400 K for siderite (Figs. 1 and 2, and Table S1). At ambient temperature, rhombohedral siderite undergoes a volume collapse at approximately 42 GPa that can be associated with an iso-symmetric spin transition, consistent with previous results^11^,^12^ (Fig. S1). However, upon laser heating to above 1400 K at approximately 50 GPa, new diffraction peaks appear, which are incompatible with the rhombohedral structure and are systematically observed at high *P-T* (Fig. 1B and Fig. S2). These peaks were also observed in the temperature-quenched samples at high pressures. Analyses of these new diffraction peaks show that they do not belong to any previously proposed high-pressure structural models of (Fe,Mg)CO_3_ including a pyroxene-like structure^13^,^14^, a monoclinic *C*2/*m* structure^15^, and a monoclinic *P*2_1_/*c* structure^16^. The diffraction patterns also differ from that for the Fe_4_(CO_4_)_3_ phase which was reported to be temperature unquenchable at high pressures^17^. Furthermore, these peaks cannot be simply assigned to any of the previously proposed structures for products of siderite dissociation, such as FeO, Fe_2_O_3_, Fe_3_O_4_, Fe_4_O_5_, CO_2_, C (nano-diamond or graphite), or Fe_3_C, at high *P-T* and variable redox conditions^15^,^16^,^17^,^18^,^19^,^31^.

The SEM-EDX mapping of the recovered sample from 90 GPa and 2200 K exhibited homogeneous distributions of Fe, C, and O in the laser-heated area, showing no compositional dissociation and diffusion during laser heating nor in *P-T* quenching (Fig. S3). Further EDX analyses of the O/Fe ratios of the recovered sample showed a consistent O/Fe ratio of three throughout the heated area, indicating that the valence state of ferrous iron should have remained unchanged during laser heating and *P-T* quenching (Table S2). These results indicate that the high-pressure phase should have the same chemical composition as the starting siderite sample [FeCO_3_] and that dissociation or valence state change of iron in siderite did not occur in our sample at the *P-T* conditions of our study. Hereafter, this high-pressure polymorph is referred to as siderite II (or Phase II), and the rhombohedral siderite is referred to as siderite I (Phase I; or siderite for simplicity).

**Figure 3:**
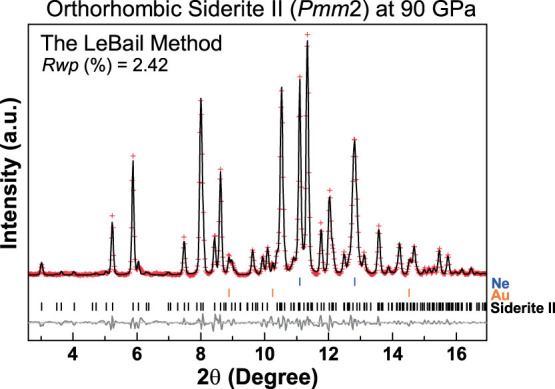
Representative LeBail fit of an X-ray diffraction spectrum of siderite II at 90 GPa and room temperature. The sample was temperature-quenched to room temperature from 2200 K at 90 GPa. Pluses: measured powder diffraction pattern after background subtraction; black solid curve: refined profile; grey solid curve: residual between the observation and the refinement; vertical ticks: Ne (blue), Au (orange), and siderite II (black).

Siderite II was initially observed upon laser heating at approximately 1400 K and 50 GPa where it co-existed with siderite I over a certain *P-T* range (Fig. 2). At *P-T* conditions higher than approximately 70 GPa and 2200 K, siderite I totally disappeared and siderite II became a single phase. X-ray diffraction patterns of the temperature-quenched siderite II were collected at 300 K in compression between 50 and 120 GPa and in decompression down to 15 GPa; below 15 GPa, siderite II became amorphous. All diffraction peaks of the siderite II can be well indexed with an orthorhombic unit cell (Fig. 1, Fig. S2, and Table S3). Based on the principles of reflection conditions for the indexed Miller indices of the diffraction peaks^32^, four potential space groups are found to be reasonable starting models for the orthorhombic structure: *P*222, *P*222_1_, *Pmm*2, and *Pmmm*. Using the GSAS software package^33^, representative LeBail refinements of the XRD patterns at 90 GPa and 300 K showed that the *Pmm*2 space group for the siderite II structural model gave the best fit with the smaller residues to the experimental spectra (Fig. 3). The *Pmm*2 structural model for siderite II contains 12 formula units in the primitive cell (Z); at a representative pressure of 90 GPa, its unit cell parameters are *a* = 10.9902 (±0.0028) Å, *b* = 6.3405 (±0.0021) Å, and *c* = 5.2726 (±0.0009) Å (Table S3). We note that previously proposed monoclinic and orthorhombic structural models of the high-pressure magnesite phase contain 12 and 6 formula units in the primitive cell, respectively^10^,^14^,^15^,^16^. We had also checked previously reported high-pressure phases of other carbonates and did not find any of them consistent with this siderite II structural model (see Table S4). It is worth noting that our observed X-ray diffraction patterns are consistent with that for the high-pressure phase of magnesite by Isshiki et al.^10^, although our proposed orthorhombic structural model has a larger unit cell than that proposed by Isshiki et al.^10^ The differences here were due to the use of high-quality diffraction peaks, including 4 diffraction peaks with *d*-spacing greater than 3.0 Å. The high-quality X-ray diffraction patterns with extended *d*-spacing range (30 peaks in total) permit us to resolve the crystal structure of the siderite II phase reliably. On the other hand, Boulard et al.^16^ proposed the monoclinic high-pressure phase of magnesite and magnesiosiderite as a result of chemical dissociation in laser-heated carbonates. These experiments were conducted without any pressure-transmitting medium in the sample chamber^16^, which may contribute to the differences in the observed phases.

It should be noted that our diffraction results do not permit the use of the Rietveld full-profile refinement to refine the carbon atom positions as well as the C-O bonding geometry and characters, because the relatively light carbon atoms do not contribute to X-ray diffraction signals significantly enough for such an analysis^34^. It has been reported that high-pressure phases of carbonates can contain tetrahedrally-coordinated carbon (CO_4_)^4−^ units with the *sp*^3^ bonding characters^15^,^16^,^35^ or the coexistence of both trigonally and tetrahedrally coordinated units^36^, instead of the trigonally-coordinated CO_3_^2−^ units alone in the siderite I. The C-O bonding characters of our observed siderite II at high pressures thus remain to be further investigated using other more sensitive methods.

### Pressure-Volume (*P-V*) Relations in Siderite I and II Phases

**Figure 4:**
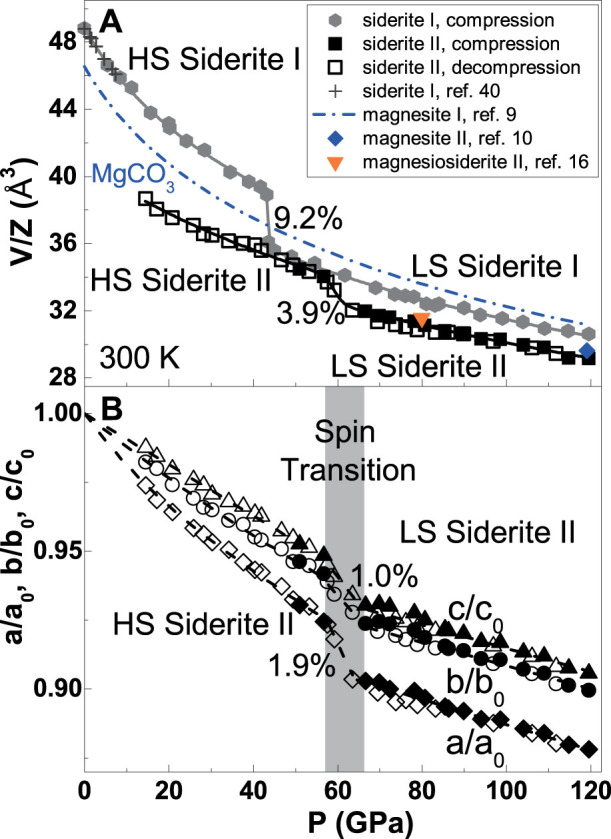
Comparison of the pressure-volume relations in the (Mg,Fe)CO_3_ phases. (*A*) Unit cell volume of siderite I and II phases as a function of pressure at ambient temperature. The vertical axis is plotted as the unit cell volume per formula unit (*V*/*Z*). The number of molecules per unit cell (*Z*) is 6 for siderite I and 12 for siderite II. HS: high-spin state; LS: low-spin state. Solid curves: modeled BM EoS fits of the experimental results. The volume collapse of 9.2 (±0.5)% and 3.9 (±0.4)% for siderite I and II can be associated with their respective spin transition at high pressures. Solid diamond: *V*/*Z* of magnesite II re-calculated from Isshiki et al.^10^ (Table S5); Solid triangle: *V*/*Z* of the high-pressure phase of magnesiosiderite [(Mg_0.25_Fe_0.75_)CO_3_] re-calculated from Boulard et al.^16^ (Table S6). (*B*) Lattice parameters of the siderite II as a function of pressure at 300 K. The lattice collapse in siderite II is 1.9%, 1.0%, and 1.0% for *a*/*a*_0_, *b*/*b*_0_, and *c*/*c*_0_, respectively, at approximately 60 GPa. Filled symbols: compression; open symbols: decompression.

Here we have used the *Pmm*2 structural model of siderite II to re-index previous diffraction results^10^,^16^ and to discuss the *P-V* relations of siderite I and II phases in the (Mg,Fe)CO_3_ system at high pressures. For re-indexing the literature data, our high-pressure orthorhombic structural model and the dichotomy method were used to obtain the lattice parameter by minimizing the *d*_Obs_-*d*_Calc_ values in our calculations. Previously proposed monoclinic or orthorhombic structural model was not used here. The *Pmm*2 structural model can be used to index previous high-pressure X-ray diffraction patterns in the (Mg,Fe)CO_3_ system including magnesite II (ref. 10) and the high-pressure phase of magnesiosiderite [(Mg_0.25_Fe_0.75_)CO_3_] (ref. 16). Using the orthorhombic structure model of siderite II, the uncertainty between the observed and calculated *d* spacings can be fairly improved by re-indexing the experimental data for magnesite II at 119 GPa and 300 K from Ref. 10 and the high-pressure phase of magnesiosiderite at 80 GPa and 300 K from Ref. 16 (Tables S5 and S6). Despite the variation in the FeCO_3_ content in these previous studies, these re-indexed unit cell parameters at high pressures are generally consistent with our experimental *P-V* results for the siderite II structural model (Fig. 4).

Examinations of the *P-V* curve of siderite I show a sharp volume reduction of 9.2 (±0.5)% at approximately 42 GPa and 300 K, that can be associated with the previously reported spin transition in the (Mg,Fe)CO_3_ system^11^,^12^ (Fig. 4). Across the spin transition, the volume of the low-spin siderite I becomes smaller than the end-member magnesite via Fe-O bond distance shrinkage in the octahedral site^37^, indicating that the effective ionic radius of Fe^2+^ in the low-spin configuration is smaller than that of Mg^2+^ (refs. 11,29). The volume difference between the low-spin siderite I and magnesite I is ~3.6% at 45 GPa and ~2.1% at 120 GPa, indicating that the low-spin siderite I is more incompressible than the end-member magnesite. Such a volume reduction related to Fe spin transition increases with increasing the molar concentration of FeCO_3_ in the (Mg,Fe)CO_3_ system^12^,^27^.

Our *P-V* results for siderite II also show an abrupt change of 3.9 (±0.4)% in the unit cell volume at approximately 60 GPa (Fig. 4A). Since there's no evidence of any further structural changes in our diffraction patterns, one can thus relate this volume change to an iso-symmetric electronic transition of Fe in siderite II from the low-spin state to the high-spin state in decompression. This spin transition for siderite II occurs at 60 GPa which is approximately 18 GPa higher than that in siderite I at 42 GPa. We note that the pressure range of the spin transition in siderite II was also where the structural transition from siderite I to siderite II was observed. The low-spin siderite II is approximately 4.5% smaller in the molar volume than the low-spin siderite I, while the high-spin siderite II is approximately 11.4% smaller than the high-spin siderite I. Furthermore, the unit cell volume of the low-spin siderite II is approximately 2% smaller than that of magnesite II at 119 GPa and 300 K and approximately 1% smaller than that of the magnesiosiderite II [(Mg_0.25_Fe_0.75_)CO_3_] at 80 GPa and 300 K (Fig. 4A). Compared with the 9.2% volume reduction across the spin transition in siderite I, the smaller volume reduction of 3.9% in siderite II is consistent with the denser structure of the high-pressure siderite II phase. Fitting the *P-V* results to the third-order Birch-Murnaghan equation of state (EoS)^38^ shows that the low-spin siderite II exhibits a higher incompressibility than the high-spin siderite II as well as the low-spin siderite I (Tables S7 and S8). Specifically, the low-spin siderite II exhibits an isothermal bulk modulus (*K*_0*T*_) of 251 (±17) GPa with a fixed pressure derivative of the *K*_0*T*_ (*K*_0*T*_') at 4, while the high-spin siderite II has a *K*_0*T*_ of 222 (±13) GPa with a fixed *K*_0*T*_' of 4.

The lattice parameters of siderite II also exhibit abrupt changes across the pressure-induced spin transition (Fig. 4B); the axial length of the *a* axis is reduced by 1.9% which is almost twice as much as that in the *b* and *c* axes. In contrast, the axial lengths of the *a* and *c* axes in siderite I are reduced by approximately 2.0% and 3.0%, respectively, across the spin crossover^12^. The difference in the changes of the unit cell parameters across the spin transition in siderite I and II likely indicates relatively denser packing structure and less compressible Fe-O bonding characters in the high-pressure phase II (See Table S8 for the EoS parameters of the siderite I and II).

### Phase Diagram of the (Mg,Fe)CO_3_ Carbonates at High Pressure and Temperature

**Figure 5:**
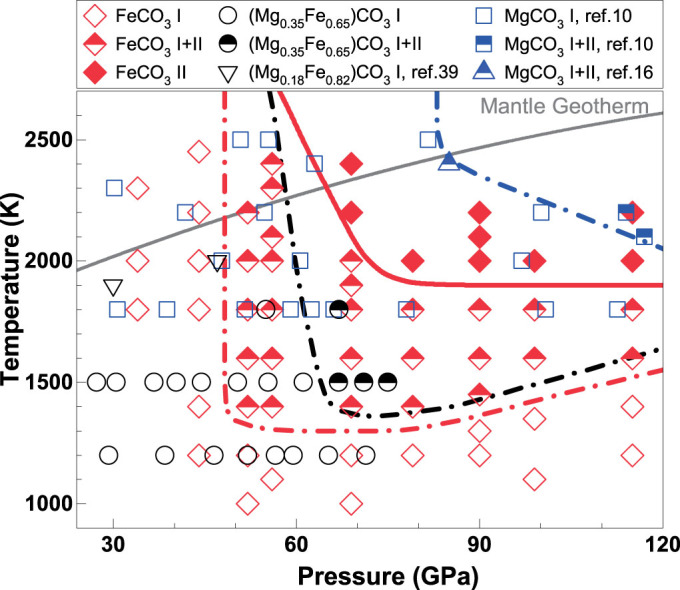
Experimentally observed phases in the (Mg,Fe)CO_3_ system at high *P-T*. Open, half-filled, and solid diamonds: phase I, I+II, and II, respectively, for siderite [FeCO_3_] (this study); open and half-filled squares: phase I and I+II, respectively, for magnesite [MgCO_3_] (ref. 10); half-filled triangle: phase I and II for magnesite^16^; open and half-filled circles: phase I and I+II, respectively, for (Mg_0.35_Fe_0.65_)CO_3_ (this study); open triangles: phase I for magnesiosiderite [(Mg_0.09_Fe_0.82_Ca_0.09_)CO_3_] (ref. 39). Red, black, and blue curves: phase transformation boundary between phase I and II for siderite, magnesiosiderite, and magnesite, respectively. Gray solid curve: expected lower-mantle geotherm^41^.

Together with previous studies^10^,^16^,^27^,^39^,^40^, here we use our experimental results on FeCO_3_ and (Fe_0.65_Mg_0.35_)CO_3_ to decipher the compositional and spin transition effects on the *P-T* phase diagram of the (Mg,Fe)CO_3_ system (Fig. 5). Analyses of the *P-T* conditions for the occurrence of phase II in the (Mg,Fe)CO_3_ system show that siderite II started to occur at 50 GPa and 1400 K, magnesiosiderite II [(Fe_0.65_Mg_0.35_)CO_3_] at 60 GPa and 1500 K, and magnesite II at 85 GPa and 2400 K. This indicates a strong effect of Fe-Mg substitution on the transitional pressure from the rhombohedral phase I to the orthorhombic phase II and that the high-pressure phase II in the FeCO_3_-rich part of the system occurs at lower *P-T* conditions than that in the MgCO_3_-rich counterpart (Fig. 5). Previous high *P-T* studies on the system have shown that the spin transition in phase I occurred sharply at approximately 45 GPa and 300 K (refs. 11,12) and that the spin crossover broadened and shifted toward higher pressures at elevated temperatures up to 1200 K (ref. 27). Because the FeO_6_ octahedra in the system are well isolated from each other by the rigid CO_3_ units, the spin transition pressure is shown to be not affected by the amount of FeCO_3_ in the system^27^; there has been no observable compositional effect on the spin transition pressure in the MgCO_3_-FeCO_3_ system^11^,^12^,^27^. Due to the iron substitution, however, the FeCO_3_-rich part of the system in the low-spin state has a smaller unit cell volume and a higher density than the MgCO_3_-rich high-spin counterpart, effectively reducing the pressure (energy) needed to transform to the high-pressure phase II by as much as approximately 35–40 GPa between the FeCO_3_ and MgCO_3_ end-members. Considering the structural transition pressures and the occurrence of the spin transition in both phase I and II, it is evident that the combination of the FeCO_3 _substitution and the spin transition of iron in the MgCO_3_-FeCO_3_ system promotes the occurrences of the high-pressure low-spin phase II at much lower *P-T* conditions than the MgCO_3_-rich counterpart^30^.

### Implications for the Deep-Mantle Carbon Storage

Spin transitions of iron in candidate mantle minerals such as perovskite [(Mg,Fe)SiO_3_] and ferropericlase [(Mg,Fe)O] have been observed to affect their physical and chemical properties including density, sound velocities, iron partitioning coefficient, as well as transport properties^22^,^23^,^24^. Our results here show that the spin transition in (Mg,Fe)CO_3_ can affect the phase diagram of the system by stabilizing the high-pressure phase II in the orthorhombic structure to lower *P-T* conditions (Figs. 2 and 5). Previous studies have indicated that magnesite likely contains approximately 15 at% iron in the Earth's mantle^7^,^21^. Along an expected lower-mantle geotherm^41^, the high-spin ferromagnesite containing 15% iron [(Mg_0.85_Fe_0.15_)CO_3_] would start to transform to the low-spin state at approximately 55 GPa and 2200 K (approximately 1400 km depth) and eventually to the ferromagnesite II phase at 80 GPa and 2400 K (approximately 1900 km depth) (Figs. 2 and 5). That is, the ferromagnesite II phase in the lower part of the lower mantle likely assumes the low-spin state. Since the low-spin iron ion is energetically more stable at high pressures than its high-spin counterpart, iron ions would partition favorably into the low-spin phase with respect to the high-spin phases in order to minimize the Gibbs free energy of the system in the lower mantle^42^. A recent experimental study shows that the partition coefficient of iron between lower-mantle silicate perovskite (Pv) and ferropericlase (Fp) [*K_D_* = (Fe/Mg)_Pv_/(Fe/Mg)_Fp_] decreases from 0.85 to 0.52 at approximately 40–47 GPa and 2000 K, which corresponds to the expected pressure-temperature conditions for the spin transition zone of ferropericlase in the lower mantle^22^. That is, the low-spin ferropericlase becomes enriched in Fe^2+^ as a result of the spin transition^23^,^42^. Considering the volume reduction across the spin transition in ferromagnesite, it is conceivable that iron would preferentially partition into the low-spin ferromagnesite II with respect to other surrounding high-spin phases in the lower mantle.

Depending on the oxygen fugacity and pressure-temperature (*P-T*) conditions, carbon can exist in various forms, including carbides, diamond, graphite, hydrocarbons, CO_2_, and carbonates, in the Earth's interior^43^. Subducted slabs have been proposed to play a certain role in transporting a certain amount of carbon into the deep lower mantle^4^,^7^. Among the transported carbon-bearing materials, ferromagnesite likely can survive the extreme *P-T* conditions of the subducting slabs in the mantle and becomes a stable carbon-bearing phase in the lower mantle^8^,^10^,^13^. Such a transport mechanism for delivering carbon into the Earth's lower mantle is also supported by the observations of the ferromagnesian carbonate inclusions in eclogite xenoliths from the deep mantle^44^ as well as magnesite-bearing inclusions in natural deep-mantle diamonds^45^,^46^; though, several previous studies have suggested that polymorphs of (Mg,Fe)CO_3_ can be replaced by the occurrence of the reduced forms of carbon such as diamond and iron carbides depending on redox conditions of the mantle regions^3^,^7^,^47^. Although the oxygen fugacity of the subducting slab materials may be not compatible with the stability of carbonates or carbonate-rich liquid^48^, the observation of carbonate inclusions in diamonds potentially brought up to the Earth's surface from the deep mantle indicates that carbonates can exist in the mantle at least locally. Future studies are needed to further understand the role of oxygen fugacity on the stability of the high-pressure ferromagnesite at relevant conditions of the lower mantle. Our results here show that ferromagnesite inside the relative cold and oxidizing slabs likely undergoes an electronic spin transition to the low-spin ferromagnesite at the mid-lower mantle conditions and then becomes stable in the orthorhombic *Pmm2* structure in the low-spin state toward the lower part of the lower mantle. It is thus conceivable that the low-spin orthorhombic ferromagnesite is a major carbon host in the deep lower mantle.

## Methods

Natural single-crystal specimens of siderite (no. NMNH R11313) from the Tsumeb Mine (Namibia) were obtained from the Department of Mineral Sciences, Smithsonian Institution^40^. Based on the wavelength dispersive spectrometer analyses (WDS) of an electron microprobe (JEOL JXA-8200), the sample has a composition of FeCO_3_ with a very minor content of MnCO_3_ (<0.2 mol%); for simplicity, the composition of this sample is referred to as FeCO_3_ by neglecting the very minor manganese content in the formula. Synchrotron X-ray diffraction patterns showed the lattice parameters of the sample to be *a* = 4.6909 (±0.0005) Å and *c* = 15.3687 (±0.0049) Å under ambient conditions. The magnesiosiderite sample was obtained from the Vargas Gem and Mineral Collection at the University of Texas at Austin (collection number: V3817), and has a chemical composition of (Fe_0.65_Mg_0.33_Mn_0.02_)CO_3_; for simplicity, this sample is presented as (Mg_0.35_Fe_0.65_)CO_3_, with the minor Mn content counted toward the total Mg content^12^,^27^. Synchrotron X-ray diffraction patterns showed the lattice parameters of the sample to be *a* = 4.6753(±0.0012) Å and *c* = 15.2794 (±0.0030) Å under ambient conditions.

Each sample was ground to micro-sized powder and then mixed with 3 wt% of micro-sized Au powder as the pressure calibrant^49^; the use of the inert Au also helped avoid potential chemical reactions with the iron-bearing sample^27^. The mixture was then slightly pressed to form a platelet approximately 10 µm thick. A small platelet with a diameter of approximately 60 μm was loaded into the sample chamber of a symmetric DAC having a pair of 200 µm flat anvils or 150–300 µm beveled anvils. Neon or dried NaCl layers were also loaded with the sample in the sample chamber and used as the thermal insulator and pressure medium. To avoid any potential air and moisture contamination in the sample chamber, the whole DAC was evacuated at 10^−2^ mbar for 30 minutes before the sample chamber was closed in a vacuum using the high-pressure gas loading system in the Mineral Physics Laboratory of the University of Texas at Austin. High-pressure synchrotron X-ray diffraction experiments were conducted at the 13IDD beamline of the GSECARS of the Advanced Photon Source (APS), Argonne National Laboratory (ANL) using a focused monochromatic X-ray beam with a wavelength (*λ*) of 0.3344 Å and a beamsize of 2 μm in diameter. For the laser heating, two infrared laser beams were focused down to 25 μm diameter on both sides of the sample, and were co-axially aligned with the incoming X-ray beam using the X-ray induced luminescence on NaCl or the sample^50^. Temperatures of the laser-heated sample were measured using thermal radiation spectra fitted to the blackbody radiation function^50^. We note that the siderite sample absorbed the infrared laser very efficiently and evenly on both sides, resulting in temperature uncertainties typically less than 50 K at high pressures. Pressure was measured through the thermal equation of state of Au at high temperature^49^. The lattice parameters were determined from the diffraction patterns processed by using the LeBail method with the GSAS software package^33^. Electron microprobe analyses of the quenched samples were conducted using the Environmental Scanning Electron Microscope (FEI Quanta 650 ESEM) and the energy dispersive spectroscopy (Bruker XFlash Detector 5010) with the electron beam resolution of 2.5 nm at 30 kV at the Texas Materials Institute of the University of Texas at Austin. The FEI Quanta 650 ESEM analyses did not require the use of surface conductive coating (such as gold or carbon), which helped preserve original characteristics of the sample especially for carbon analyses. Note that this ESEM does not have capability to do quantitative analysis on carbon concentrations, instead of qualitative determination.

## Author Contributions

J.F.L. & J.L. conceived & designed the research; J.L., J.F.L., V.B.P. conducted the experiments; J.L. & J.F.L. wrote the paper. All authors reviewed the manuscript.

## Supplementary Material

Supplementary Information SI

## References

[b1] Manning C. E., Shock E. L. & Sverjensky D. A. The chemistry of carbon in aqueous fluids at crustal and upper-mantle conditions: experimental and theoretical constraints. Rev. Mineral. Geochem. 75, 109–148, 10.2138/rmg.2013.75.5 (2013).

[b2] Dasgupta R. & Hirschmann M. M. Melting in the Earth's deep upper mantle caused by carbon dioxide. Nature 440, 659–662 (2006). 16572168 10.1038/nature04612

[b3] Rohrbach A. & Schmidt M. W. Redox freezing and melting in the Earth's deep mantle resulting from carbon-iron redox coupling. Nature 472, 209–212, 10.1038/nature09899 (2011). 21441908

[b4] Walter M. J. *et al.* Deep mantle cycling of oceanic crust: evidence from diamonds and their mineral inclusions. Science 334, 54–57, 10.1126/science.1209300 (2011). 21921159

[b5] Jana D. & Walker D. The impact of carbon on element distribution during core formation. Geochim. Cosmochim. Acta 61, 2759–2763, 10.1016/S0016-7037(97)00091-4 (1997).

[b6] Hazen R. M. & Schiffries C. M. Why deep carbon? Rev. Mineral. Geochem. 75, 1–6, 10.2138/rmg.2013.75.1 (2013).

[b7] Dasgupta R. & Hirschmann M. M. The deep carbon cycle and melting in Earth's interior. Earth Planet. Sci. Lett. 298, 1–13, 10.1016/j.epsl.2010.06.039 (2010).

[b8] Biellmann C., Gillet P., Guyot F. o., Peyronneau, J. & Reynard, B. Experimental evidence for carbonate stability in the Earth's lower mantle. Earth Planet. Sci. Lett. 118, 31–41, 10.1016/0012-821x(93)90157-5 (1993).

[b9] Fiquet G. *et al.* Structural refinements of magnesite at very high pressure. Am. Mineral. 87, 1261–1265 (2002).

[b10] Isshiki M. *et al.* Stability of magnesite and its high-pressure form in the lowermost mantle. Nature 427, 60–63 (2004). 14702083 10.1038/nature02181

[b11] Lavina B. *et al.* Siderite at lower mantle conditions and the effects of the pressure-induced spin-pairing transition. Geophys. Res. Lett. 36, L23306, 10.1029/2009gl039652 (2009).

[b12] Lin J.-F., Liu J., Jacobs C. & Prakapenka V. B. Vibrational and elastic properties of ferromagnesite across the electronic spin-pairing transition of iron. Am. Mineral. 97, 583–591, 10.2138/am.2012.3961 (2012).

[b13] Panero W. R. & Kabbes J. E. Mantle-wide sequestration of carbon in silicates and the structure of magnesite II. Geophys. Res. Lett. 35, L14307, 10.1029/2008GL034442 (2008).

[b14] Skorodumova N. V., Belonoshko A. B., Huang L., Ahuja R. & Johansson B. Stability of the MgCO_3_ structures under lower mantle conditions. Am. Mineral. 90, 1008–1011, 10.2138/am.2005.1685 (2005).

[b15] Oganov A. R., Ono S., Ma Y., Glass C. W. & Garcia A. Novel high-pressure structures of MgCO_3_, CaCO_3_ and CO_2_ and their role in Earth's lower mantle. Earth Planet. Sci. Lett. 273, 38–47, 10.1016/j.epsl.2008.06.005 (2008).

[b16] Boulard E. *et al.* New host for carbon in the deep Earth. Proc. Natl. Acad. Sci. USA 108, 5184–5187 (2011). 21402927 10.1073/pnas.1016934108 PMC3069163

[b17] Boulard E. *et al.* Experimental investigation of the stability of Fe-rich carbonates in the lower mantle. J. Geophys. Res. 117, B02208, 10.1029/2011jb008733 (2012).

[b18] Gallagher P. K. & Warne S. S. J. Thermomagnetometry and thermal decomposition of siderite. Thermochim. Acta 43, 253–267, 10.1016/0040-6031(81)85183-0 (1981).

[b19] Tao R., Fei Y. & Zhang L. Experimental determination of siderite stability at high pressure. Am. Mineral. 98, 1565–1572, 10.2138/am.2013.4351 (2013).

[b20] Frost D. J. & McCammon C. A. The redox state of Earth's mantle. Annu. Rev. Earth Planet. Sci. 36, 389–420, 10.1146/annurev.earth.36.031207.124322 (2008).

[b21] Dasgupta R., Hirschmann M. M. & Withers A. C. Deep global cycling of carbon constrained by the solidus of anhydrous, carbonated eclogite under upper mantle conditions. Earth Planet. Sci. Lett. 227, 73–85, 10.1016/j.epsl.2004.08.004 (2004).

[b22] Irifune T. *et al.* Iron partitioning and density changes of pyrolite in Earth's lower mantle. Science 327, 193–195 (2010). 19965719 10.1126/science.1181443

[b23] Lin J.-F., Speziale S., Mao Z. & Marquardt H. Effects of the electronic spin transitions of iron in lower mantle minerals: implications for deep mantle geophysics and geochemistry. Rev. Geophys. 51, 244–275, 10.1002/rog.20010 (2013).

[b24] Mao Z., Lin J.-F., Liu J. & Prakapenka V. B. Thermal equation of state of lower-mantle ferropericlase across the spin crossover. Geophys. Res. Lett. 38, L23308, 10.1029/2011gl049915 (2011).

[b25] Oganov A. R., Hemley R. J., Hazen R. M. & Jones A. P. Structure, bonding, and mineralogy of carbon at extreme conditions. Rev. Mineral. Geochem. 75, 47–77, 10.2138/rmg.2013.75.3 (2013).

[b26] Mattila A. *et al.* Pressure induced magnetic transition in siderite FeCO_3_ studied by x-ray emission spectroscopy. J. Phys. Condens. Matter 19, 386206 (2007).

[b27] Liu J., Lin J.-F., Mao Z. & Prakapenka V. B. Thermal equation of state and spin transition of magnesiosiderite at high pressure and temperature. Am. Mineral. 99, 84–93, 10.2138/am.2014.4553 (2014).

[b28] Shi H., Luo W., Johansson B. & Ahuja R. First-principles calculations of the electronic structure and pressure-induced magnetic transition in siderite FeCO_3_. Phys. Rev. B 78, 155119 (2008).

[b29] Shannon R. D. Revised effective ionic radii and systematic studies of interatomic distances in halides and chalcogenides. Acta Crystallogr. Sect. A 32, 751–767, 10.1107/S0567739476001551 (1976).

[b30] Hazen R. M., Downs R. T. & Prewitt C. T. Principles of comparative crystal chemistry. Rev. Mineral. Geochem. 41, 1–33, 10.2138/rmg.2000.41.1 (2000).

[b31] Lavina B. *et al.* Discovery of the recoverable high-pressure iron oxide Fe_4_O_5_. Proc. Natl. Acad. Sci. USA 108, 17281–17285, 10.1073/pnas.1107573108 (2011). 21969537 PMC3198347

[b32] Looijenga-Vosa A. & Buerger M. J. in International Tables for Crystallography Vol A. (ed Theo Hahn) Ch. 3.1, 46–51 (Springer, 2006).

[b33] Toby B. EXPGUI, a graphical user interface for GSAS. J. Appl. Cryst. 34, 210–213, 10.1107/S0021889801002242 (2001).

[b34] McCusker L. B., Von Dreele R. B., Cox D. E., Louer D. & Scardi P. Rietveld refinement guidelines. J. Appl. Cryst. 32, 36–50, 10.1107/S0021889898009856 (1999).

[b35] Arapan S., Souza de Almeida J. & Ahuja R. Formation of *sp*^3^ hybridized bonds and stability of CaCO_3_ at very high pressure. Phys. Rev. Lett. 98, 268501 (2007). 17678133 10.1103/PhysRevLett.98.268501

[b36] Merlini M. *et al.* Structures of dolomite at ultrahigh pressure and their influence on the deep carbon cycle. Proc. Natl. Acad. Sci. USA 109, 13509–13514, 10.1073/pnas.1201336109 (2012). 22869705 PMC3427128

[b37] Lavina B. *et al.* Structure of siderite FeCO_3_ to 56 GPa and hysteresis of its spin-pairing transition. Phys. Rev. B 82, 064110 (2010).

[b38] Birch F. Finite strain isotherm and velocities for single-crystal and polycrystalline NaCl at high pressures and 300 K. J. Geophys. Res. 83, 1257–1268, 10.1029/JB083iB03p01257 (1978).

[b39] Santillan J. & Williams Q. A high-pressure infrared and X-ray study of FeCO_3_ and MnCO_3_: comparison with CaMg(CO_3_)_2_-dolomite. Phys. Earth Planet. Inter. 143–144, 291–304, 10.1016/j.pepi.2003.06.007 (2004).

[b40] Zhang J., Martinez I., Guyot F. & Reeder R. J. Effects of Mg-Fe^2+^ substitution in calcite-structure carbonates: thermoelastic properties. Am. Mineral. 83, 280–287 (1998).

[b41] Katsura T., Yoneda A., Yamazaki D., Yoshino T. & Ito E. Adiabatic temperature profile in the mantle. Phys. Earth Planet. Inter. 183, 212–218, 10.1016/j.pepi.2010.07.001 (2010).

[b42] Badro J., Fiquet G. & Guyot F. in *Earth*'*s Deep Mantle: Structure, Composition, and Evolution* Vol. 160 *Geophysical Monograph Series* (eds Robert D. van der Hilst, Jay D. Bass, Jan Matas, & Jeannot Trampert) 241–260 (AGU., 2005).

[b43] Palyanov Y. N. *et al.* Mantle–slab interaction and redox mechanism of diamond formation. Proc. Natl. Acad. Sci. USA 110, 20408–20413, 10.1073/pnas.1313340110 (2013). 24297876 PMC3870714

[b44] Liou J. G., Zhang R. Y., Ernst W. G., Rumble D. & Maruyama S. High-pressure minerals from deeply subducted metamorphic rocks. Rev. Mineral. Geochem. 37, 33–96 (1998).

[b45] Wang A., Pasteris J. D., Meyer H. O. A. & Dele-Duboi M. L. Magnesite-bearing inclusion assemblage in natural diamond. Earth Planet. Sci. Lett. 141, 293–306, 10.1016/0012-821X(96)00053-2 (1996).

[b46] Berg G. W. Evidence for carbonate in the mantle. Nature 324, 50–51 (1986).

[b47] Dasgupta R. Ingassing, Storage, and Outgassing of Terrestrial Carbon through Geologic Time. Rev. Mineral. Geochem. 75, 183–229, 10.2138/rmg.2013.75.7 (2013).

[b48] Stagno V., Ojwang D. O., McCammon C. A. & Frost D. J. The oxidation state of the mantle and the extraction of carbon from Earth's interior. Nature 493, 84–88, 10.1038/nature11679 (2013). 23282365

[b49] Fei Y. *et al.* Toward an internally consistent pressure scale. Proc. Natl. Acad. Sci. USA 104, 9182–9186, 10.1073/pnas.0609013104 (2007). 17483460 PMC1890468

[b50] Prakapenka V. B. *et al.* Advanced flat top laser heating system for high pressure research at GSECARS: application to the melting behavior of germanium. High Pressure Res. 28, 225–235, 10.1080/08957950802050718 (2008).

