# Supplementary material for: High-Pressure Orthorhombic Ferromagnesite as a Potential Deep-Mantle Carbon Carrier
Source: Sci Rep. 2015 Jan 6;5:7640. doi: 10.1038/srep07640 (PMC4284511; doi:10.1038/srep07640)
Supplement: Supplementary Information — SI [file srep07640-s1.pdf]

Supplementary Material

High-Pressure Orthorhombic Ferromagnesite as a Potential Deep-Mantle Carbon Carrier

Jin Liu[a], Jung-Fu Lin[a,b], and Vitali B. Prakapenka[c]

[a]Department of Geological Sciences, Jackson School of Geosciences, The University of Texas at Austin, Austin, TX 78712, USA

[b]Center for High Pressure Science and Technology Advanced Research (HPSTAR), Shanghai 201203, People's Republic of China

[c]Consortium for Advanced Radiation Sources, The University of Chicago, Chicago, IL 60637, USA

Supplementary Text

Indexing the powder diffraction patterns. The dichotomy method[1] has been used for indexing the powder diffraction patterns of the high-pressure phase siderite II (Table S3). It consists of finding the unit-cell parameters from the *d*-spacing values available in a diffraction pattern[1]. Literature data on magnesite II (ref. 2) and magnesiosiderite II (ref. 3) have also been re-indexed using the current orthorhombic siderite II structural model (Tables S5 and S6). The derived unit cell parameters are consistent within the high-pressure phase of the magnesite-siderite system.

Equation of state parameters. The third-order Birch-Murnaghan equation of state[4] was used to fit to the pressure-volume data of the siderite I and II, respectively, at 300 K:

$$P = \frac{3K_{0T}}{2}\left[\left(\frac{V_{0T}}{V}\right)^{\frac{7}{3}} - \left(\frac{V_{0T}}{V}\right)^{\frac{5}{3}}\right]\left\{1 + \frac{3}{4}\left(K_{0T}^{'} - 4\right)\left[\left(\frac{V_{0T}}{V}\right)^{\frac{2}{3}} - 1\right]\right\}, \quad \text{[S1]}$$

where P is pressure, V is the unit-cell volume, K_{0T} is the isothermal bulk modulus at ambient pressure and K_{0T}' is its pressure derivative, and $0T$ denotes ambient pressure and a given temperature, respectively. The derived incompressibility for siderite is consistent with the reported value for siderite (see Table S8).

Supplementary References

1. Boultif, A. & Louer, D. Powder pattern indexing with the dichotomy method. *J. Appl. Cryst.* **37**, 724-731, doi:10.1107/S0021889804014876 (2004).
2. Isshiki, M. *et al.* Stability of magnesite and its high-pressure form in the lowermost mantle. *Nature* **427**, 60-63 (2004).
3. Boulard, E. *et al.* New host for carbon in the deep Earth. *Proc. Natl. Acad. Sci. USA* **108**, 5184-5187 (2011).
4. Birch, F. Finite strain isotherm and velocities for single-crystal and polycrystalline NaCl at high pressures and 300 K. *J. Geophys. Res.* **83**, 1257-1268, doi:10.1029/JB083iB03p01257 (1978).
5. Fei, Y. *et al.* Toward an internally consistent pressure scale. *Proc. Natl. Acad. Sci. USA* **104**, 9182-9186, doi:10.1073/pnas.0609013104 (2007).
6. Skorodumova, N. V., Belonoshko, A. B., Huang, L., Ahuja, R. & Johansson, B. Stability of the $MgCO_3$ structures under lower mantle conditions. *Am. Mineral.* **90**, 1008-1011, doi:10.2138/am.2005.1685 (2005).
7. Oganov, A. R., Ono, S., Ma, Y., Glass, C. W. & Garcia, A. Novel high-pressure structures of $MgCO_3$, $CaCO_3$ and CO_2 and their role in Earth's lower mantle. *Earth Planet. Sci. Lett.* **273**, 38-47, doi:10.1016/j.epsl.2008.06.005 (2008).
8. Oganov, A. R., Glass, C. W. & Ono, S. High-pressure phases of $CaCO_3$: Crystal structure prediction and experiment. *Earth Planet. Sci. Lett.* **241**, 95-103, doi:10.1016/j.epsl.2005.10.014 (2006).

9. Merrill, L. & Bassett, W. A. The crystal structure of $CaCO_3$(II), a high-pressure metastable phase of calcium carbonate. *Acta Crystallogr. Sect. B* **31**, 343-349, doi:10.1107/S0567740875002774 (1975).

10. Arapan, S. & Ahuja, R. High-pressure phase transformations in carbonates. *Phys. Rev. B* **82**, 184115 (2010).

11. Ono, S., Kikegawa, T., Ohishi, Y. & Tsuchiya, J. Post-aragonite phase transformation in $CaCO_3$ at 40 GPa. *Am. Mineral.* **90**, 667-671, doi:10.2138/am.2005.1610 (2005).

12. Ono, S. High-pressure phase transformation in $MnCO_3$: a synchrotron XRD study. *Mineral. Mag.* **71**, 105-111, doi:10.1180/minmag.2007.071.1.105 (2007).

13. Merlini, M., Hanfland, M. & Crichton, W. A. $CaCO_3$-III and $CaCO_3$-VI, high-pressure polymorphs of calcite: Possible host structures for carbon in the Earth's mantle. *Earth Planet. Sci. Lett.* **333–334**, 265-271, doi:10.1016/j.epsl.2012.04.036 (2012).

14. Ono, S., Kikegawa, T. & Ohishi, Y. High-pressure transition of $CaCO_3$. *Am. Mineral.* **92**, 1246-1249, doi:10.2138/am.2007.2649 (2007).

Supplementary Figure Captions

Fig. S1. Representative X-ray diffraction patterns of siderite I in compression up to 120 GPa at 300 K. Gold (Au) was used as the primary pressure calibrant, while NaCl (B1 or B2 structure) was served as the secondary pressure calibrant as well as the thermal insulator and pressure medium[5]. Miller indices (hkl) of siderite I are labeled as I(hkl). HS and LS states are labeled to illustrate the splitting of the diffraction peaks across the spin transition of siderite I at 42 GPa and 300 K. The wavelength of the monochromatic X-ray beam was 0.3344 Å.

Fig. S2. Representative X-ray diffraction patterns of siderite I and II phases before, during, and after laser heating at 115 GPa. Gold (Au) was used as the primary pressure calibrant, while NaCl (B1 or B2 structure) was served as the secondary pressure calibrant as well as the thermal insulator and pressure medium[5]. Miller indices (hkl) of siderite I and II are labeled as I(hkl) and II(hkl), respectively. The wavelength of the monochromatic X-ray beam was 0.3344 Å.

Fig. S3. Representative SEM-EDX images of the high *P-T* quenched $FeCO_3$ sample. The sample was heated to 2200 K at 90 GPa and then eventually decompressed and recovered at ambient conditions for the analyses. (*A*) Back-scattered electron image of the recovered sample. The dashed circles show the representative laser-heated spot where siderite II was observed in the XRD patterns. Individual SEM-EDX mappings: (*B*) Oxygen; (*C*) Carbon; (*D*) Iron.

Table S1. Experimental conditions for siderite at high pressures and temperatures. Data points were compiled using the experimental sequence. Ne medium was used in Samples #1st and #2nd, whereas NaCl medium was used in Sample #3rd. The high-pressure siderite II phase can be reproduced and observed in all three samples at high pressure-temperature conditions.

Sample	P (GPa)	T (K)	Phase(s)	Sample	P (GPa)	T (K)	Phase(s)
1st	0	300	I	2nd	69.0	1200	I
1st	5.5	300	I	2nd	69.0	1400	I+II
1st	8.5	300	I	2nd	69.0	1600	I+II
1st	11.2	300	I	2nd	69.0	1800	I+II
1st	15.7	300	I	2nd	69.0	1900	I+II
1st	20.0	300	I	2nd	69.0	2000	I+II
1st	24.2	300	I	2nd	69.0	2200	II
1st	28.5	300	I	2nd	69.0	2400	II
1st	34.1	300	I	2nd	73.5	300	I
1st	34.1	1800	I	2nd	76.4	300	I
1st	34.1	2000	I	2nd	79.2	300	I
1st	34.1	2300	I	2nd	79.2	1200	I
1st	38.8	300	I	2nd	79.2	1400	I+II
1st	42.0	300	I	2nd	79.2	1600	I+II
1st	44.2	2450	I	2nd	79.2	1800	I+II
1st	44.2	2200	I	2nd	79.2	2000	II
1st	44.2	2000	I	2nd	81.3	300	I
1st	44.2	1800	I	2nd	84.4	300	I
1st	44.2	1400	I	2nd	90.0	300	I
1st	44.2	1200	I	2nd	90.0	1200	I
1st	44.2	300	I	2nd	90.0	1300	I
1st	48.7	300	I	2nd	90.0	1450	I+II
1st	58.9	300	I	2nd	90.0	1600	I+II
1st	51.9	300	I	2nd	90.0	1800	I+II
1st	51.9	1000	I	2nd	90.0	2000	II
1st	51.9	1200	I	2nd	90.0	2100	II
1st	51.9	1400	I+II	2nd	90.0	2200	II
1st	51.9	1600	I+II	3rd	93.8	300	I
1st	51.9	1800	I+II	3rd	99.4	300	I
1st	51.9	2000	I+II	3rd	99.4	1100	I
1st	51.9	2200	I+II	3rd	99.4	1350	I
1st	56.3	300	I	3rd	99.4	1600	I+II
1st	56.3	1100	I	3rd	99.4	1800	I+II
1st	56.3	1400	I+II	3rd	99.4	2000	II
1st	56.3	1600	I+II	3rd	103.9	300	I
1st	56.3	1800	I+II	3rd	109.1	300	I
1st	56.3	2000	I+II	3rd	115.2	1200	I

1st	56.3	2100	I+II	3rd	115.2	1400	I
1st	56.3	2300	I+II	3rd	115.2	300	I
1st	56.3	2400	I+II	3rd	115.2	1600	I+II
2nd	54.6	300	I	3rd	115.2	1800	I+II
2nd	61.7	300	I	3rd	115.2	2000	II
2nd	65.3	300	I	3rd	115.2	2200	II
2nd	69.0	300	I	3rd	119.5	300	I
2nd	69.0	1000	I				

Table S2. Chemical analyses of iron and oxygen contents in the recovered sample from laser heating at 90 GPa and 2200 K. The analyses were conducted using the Environmental Scanning Electron Microscope (Model Quanta 650 FEG) and the energy dispersive spectroscopy (Bruker XFlash Detector 5010) with the electron beam resolution of 2.5 nm at 30 kV.

	1	2	3	4	5	6
O (at.%)	75.6 (±5.1)	75.1 (±5.0)	75.0 (±5.0)	76.1 (±4.6)	75.6 (±5.2)	75.7 (±5.5)
Fe (at.%)	24.4 (±2.3)	24.9 (±2.3)	25.0 (±2.3)	23.9 (±1.9)	24.4 (±2.3)	24.3 (±2.5)
O/Fe	3.0 (±0.3)	3.0 (±0.3)	3.0 (±0.3)	3.1 (±0.3)	3.0 (±0.3)	3.1 (±0.3)

Table S3. Observed and calculated *d* spacings for the siderite II phase at 90 GPa and room temperature. The orthorhombic structure with the formula unit per unit cell (*Z*) of 12 was used for the calculations with representative a = 10.9902 (±0.0028) Å, b = 6.3405 (±0.0021) Å, c = 5.2726 (±0.0009) Å, and V = 367.4 (±0.3) Å^3 via the dichotomy method[1]. d_{Obs}: observed *d* spacings of the X-ray diffraction peaks; d_{Cal}: calculated *d* spacings; d_{Obs}-d_{Calc}: difference between the observed and calculated *d* spacings; I_{Obs}: observed intensities of the diffraction peaks that have been scaled to the highest intensity as 100.

H	K	L	d_{Obs} (Å)	d_{Calc} (Å)	d_{Obs}-d_{Calc} (Å)	I_{Obs}
0	1	0	6.3387	6.3405	-0.0018	3
3	0	0	3.6634	3.6634	0.0001	21
2	1	1	3.2638	3.2623	0.0015	45
0	2	0	3.1655	3.1702	-0.0047	1
1	0	2	2.5634	2.5636	-0.0002	14
3	2	0	2.3959	2.3972	-0.0013	89
4	1	1	2.2718	2.2744	-0.0026	13
2	1	2	2.2267	2.2257	0.0010	43
3	0	2	2.1388	2.1398	-0.0010	3
1	2	2	1.9926	1.9934	-0.0008	9
1	3	1	1.9289	1.9312	-0.0023	4
2	2	2	1.9004	1.9018	-0.0014	10
4	0	2	1.9004	1.9023	-0.0019	10
2	3	1	1.8451	1.8475	-0.0024	4
4	1	2	1.8229	1.8220	0.0009	78
6	1	0	1.7595	1.7597	-0.0002	1
0	1	3	1.6934	1.6937	-0.0003	100
4	2	2	1.6315	1.6311	0.0004	15
5	1	2	1.6315	1.6314	0.0001	15
4	3	1	1.5950	1.5966	-0.0016	34
2	3	2	1.5794	1.5794	0.0001	8
0	2	3	1.5376	1.5371	0.0005	9
3	1	3	1.5376	1.5373	0.0003	9
7	1	1	1.4650	1.4641	0.0009	7
3	2	3	1.4152	1.4171	-0.0019	21
6	3	0	1.3841	1.3842	-0.0001	1
0	3	3	1.3516	1.3513	0.0003	14
1	0	4	1.3091	1.3088	0.0003	11
6	0	3	1.2691	1.2682	0.0009	1
2	1	4	1.2563	1.2564	-0.0001	1
6	1	3	1.2436	1.2435	0.0001	11
9	0	0	1.2222	1.2211	0.0011	6

Table S4. High-pressure phases of carbonates reported in previous works.

	Magnesite-Siderite [$(Mg,Fe)CO_3$]	Calcite [$CaCO_3$]	Aragonite [$CaCO_3$]	Rhodochrosite [$MnCO_3$]
Phase I	$R\bar{3}c$	$R\bar{3}c$	*Pmcn*	$R\bar{3}c$
Phase II	orthorhombic[2]<br>$P2_1/c$ (ref. 3)<br>$C2/c$ (ref. 6)<br>$C2/m$ (ref. 7)<br>$C222_1$ (ref. 8)	$P2_1/c$ (ref. 9)	*Pmmn* (refs. 8,10)<br>$P2_12_12$ (ref. 11)	orthorhombic[12]
Phase III	*Pmcn* (ref. 6)<br>$P2_1$ (ref. 7)	$P\bar{1}$ (ref. 13)<br>orthorhombic[13,14]	$C222_1$<br>(refs. 8,10,14)	
Phase IV			*Pmcn* (ref. 10)	

Table S5. Observed and calculated *d* spacings for magnesite II at 119 GPa and 300 K using the experimental data by Isshiki *et al.*[2]. The orthorhombic structural model gives magnesite II unit cell parameters of a = 10.8623 (±0.0076) Å, b = 6.2798 (±0.0043) Å, c = 5.1911 (±0.0012) Å, V = 354.1 (±0.5) Å^3, which overall show smaller uncertainties between observed and calculated *d* spacings. For re-indexing the literature data, our high-pressure orthorhombic structural model and the dichotomy method were used to obtain the lattice parameter by minimizing the d_{Obs}-d_{Calc} values in our calculations. Previously proposed monoclinic or orthorhombic structural model was not used here. d_{Obs}: observed *d* spacings of the X-ray diffraction peaks; d_{Cal}: calculated *d* spacings; d_{Obs}-d_{Calc}: difference between the observed and calculated *d* spacings. The first column of the d_{Obs}-d_{Calc} listed below represents results using our proposed orthorhombic structure for the phase II, while the second column of d_{Obs}-d_{Calc} are results from the literature using their orthorhombic structural model[2].

H	K	L	d_{Obs}* (Å)	d_{Calc} (Å)	d_{Obs}-d_{Calc} (Å)	d_{Obs}-d_{Calc}* (Å)
1	0	2	2.5189	2.5197	-0.0008	0.0050
3	2	0	2.3677	2.3680	-0.0003	-0.0046
2	1	2	2.1919	2.1907	0.0012	-0.0001
3	2	1	2.1611	2.1582	0.0028	-0.0001
1	2	2	1.9646	1.9645	0.0001	-0.0022
4	0	2	1.8728	1.8737	-0.0009	0.0022
4	1	2	1.7953	1.7954	-0.0001	0.0003
0	1	3	1.6660	1.6661	-0.0001	-0.0001

* Obtained from Isshiki *et al.*[2].

Table S6. Observed and calculated *d* spacings for the high-pressure phase II of magnesiosiderite [$(Mg_{0.25}Fe_{0.75})CO_3$] at 80 GPa and 300 K using the experimental data by Boulard *et al.*[3]. The orthorhombic structural model gives the phase II unit cell parameters of a = 11.10 (±0.02) Å, b = 6.52 (±0.01) Å, c = 5.27 (±0.02) Å, V = 381.4 (±1.7) Å^3. For re-indexing the literature data, our high-pressure orthorhombic structural model and the dichotomy method were used to obtain the lattice parameter by minimizing the d_{Obs}-d_{Calc} values in our calculations. Previously proposed monoclinic or orthorhombic structural model was not used here. d_{Obs}: observed *d* spacings of the X-ray diffraction peaks; d_{Cal}: calculated *d* spacings; d_{Obs}-d_{Calc}: difference between the observed and calculated *d* spacings. The first column of the d_{Obs}-d_{Calc} listed below represents results using our proposed orthorhombic structure for the phase II, while the second column of d_{Obs}-d_{Calc} are results from the literature using the monoclinic structural model[3].

H	K	L	d_{Obs}* (Å)	d_{Calc} (Å)	d_{Obs}-d_{Calc} (Å)	d_{Obs}-d_{Calc}* (Å)
3	0	0	3.70	3.70	0.00	0.02
1	2	0	3.15	3.14	0.01	0.00
0	0	2	2.61	2.63	-0.02	0.02
1	0	2	2.57	2.57	0.00	0.00
1	1	2	2.39	2.39	0.00	0.04
4	1	1	2.29	2.30	-0.01	0.00
2	1	2	2.26	2.24	0.02	0.00
0	3	1	2.00	2.01	-0.01	0.00
2	2	2	1.92	1.92	0.00	-0.01
6	0	0	1.85	1.85	0.00	0.00
4	1	2	1.83	1.83	0.00	0.00
0	0	3	1.76	1.76	0.00	0.00
6	1	1	1.69	1.69	0.00	-0.01
0	3	2	1.67	1.67	0.00	0.00

* Obtained from Boulard *et al.*[3].

Table S7. Lattice parameters of siderite II at high pressures and ambient temperature.

P (GPa)	*a* (Å)	*b* (Å)	*c* (Å)	*V* (Å^3)
66.6 (±1.2)	11.1483 (±0.0048)	6.4317 (±0.0021)	5.3484 (±0.0028)	383.5 (±0.3)
69.8 (±1.5)	11.1183 (±0.0068)	6.4144 (±0.0025)	5.3341 (±0.0023)	380.4 (±0.3)
72.3 (±1.5)	11.1068 (±0.0055)	6.4078 (±0.0031)	5.3285 (±0.0017)	379.2 (±0.3)
78.2 (±1.6)	11.0799 (±0.0039)	6.3923 (±0.0024)	5.3156 (±0.0037)	376.5 (±0.3)
80.7 (±1.4)	11.0490 (±0.0057)	6.3744 (±0.0036)	5.3008 (±0.0030)	373.3 (±0.4)
85.4 (±1.9)	11.0102 (±0.0058)	6.3520 (±0.0043)	5.2822 (±0.0025)	369.4 (±0.4)
89.8 (±1.7)	10.9902 (±0.0028)	6.3405 (±0.0021)	5.2726 (±0.0009)	367.4 (±0.2)
86.3 (±1.9)	11.0002 (±0.0078)	6.3463 (±0.0041)	5.2774 (±0.0038)	368.4 (±0.4)
83.2 (±2.1)[d]	11.0020 (±0.0051)	6.3473 (±0.0032)	5.2783 (±0.0022)	368.6 (±0.3)
78.9 (±1.7)[d]	11.0171 (±0.0092)	6.3560 (±0.0055)	5.2855 (±0.0036)	370.1 (±0.5)
76.2 (±1.6)[d]	11.0403 (±0.0091)	6.3694 (±0.0031)	5.2966 (±0.0027)	372.5 (±0.4)
73.8 (±1.7)[d]	11.0557 (±0.0089)	6.3783 (±0.0041)	5.3040 (±0.0032)	374.0 (±0.4)
69.5 (±1.5)[d]	11.0737 (±0.0088)	6.3887 (±0.0061)	5.3127 (±0.0034)	375.8 (±0.5)
63.5 (±1.4)[d]	11.1573 (±0.0104)	6.4369 (±0.0065)	5.3528 (±0.0044)	384.4 (±0.6)
59.2 (±1.8)[d]	11.4063 (±0.0095)	6.4824 (±0.0053)	5.3906 (±0.0033)	398.6 (±0.5)
57.5 (±1.6)[d]	11.4616 (±0.0115)	6.5138 (±0.0069)	5.4167 (±0.0046)	404.4 (±0.7)
53.3 (±1.9)[d]	11.5339 (±0.0099)	6.5548 (±0.0052)	5.4508 (±0.0044)	412.1 (±0.6)
49.5 (±1.6)[d]	11.5759 (±0.0125)	6.5787 (±0.0059)	5.4707 (±0.0056)	416.6 (±0.7)
46.7 (±1.8)[d]	11.6081 (±0.0131)	6.5970 (±0.0071)	5.4859 (±0.0042)	420.1 (±0.7)
42.0 (±1.6)[d]	11.6714 (±0.0114)	6.6330 (±0.0062)	5.5158 (±0.0055)	427.0 (±0.7)
40.4 (±1.5)[d]	11.7083 (±0.0146)	6.6540 (±0.0083)	5.5333 (±0.0046)	431.1 (±0.8)
37.6 (±1.5)[d]	11.7158 (±0.0128)	6.6582 (±0.0088)	5.5368 (±0.0052)	431.9 (±0.8)
34.2 (±1.7)[d]	11.7346 (±0.0165)	6.6689 (±0.0075)	5.5457 (±0.0079)	434.0 (±1.0)
30.3 (±1.2)[d]	11.7682 (±0.0155)	6.6880 (±0.0096)	5.5616 (±0.0055)	437.7 (±1.0)
28.2 (±1.3)[d]	11.7797 (±0.0176)	6.6945 (±0.0085)	5.5670 (±0.0039)	439.0 (±0.9)
25.9 (±1.5)[d]	11.8321 (±0.0215)	6.7243 (±0.0106)	5.5918 (±0.0121)	444.9 (±1.4)
20.9 (±1.1)[d]	11.8796 (±0.0177)	6.7513 (±0.0089)	5.6142 (±0.0075)	450.3 (±1.1)
17.1 (±0.8)[d]	11.9344 (±0.0236)	6.7825 (±0.0091)	5.6401 (±0.0099)	456.5 (±1.4)
14.5 (±0.9)[d]	11.9982 (±0.0258)	6.8187 (±0.0112)	5.6703 (±0.0125)	463.9 (±1.6)
51.0 (±1.6)*	11.5508 (±0.0085)	6.5645 (±0.0039)	5.4588 (±0.0049)	413.9 (±0.5)
56.7 (±1.5)*	11.4987 (±0.0095)	6.5349 (±0.0059)	5.4342 (±0.0064)	408.3 (±0.7)
94.0 (±1.9)*	10.9552 (±0.0103)	6.3203 (±0.0064)	5.2558 (±0.0055)	363.9 (±0.6)
98.5 (±1.8)*	10.9515 (±0.0099)	6.3182 (±0.0059)	5.2540 (±0.0066)	363.5 (±0.7)
104.0 (±2.2)*	10.9105 (±0.0115)	6.2945 (±0.0069)	5.2344 (±0.0067)	359.5 (±0.7)
108.9 (±2.1)*	10.8918 (±0.0169)	6.2837 (±0.0097)	5.2254 (±0.0075)	357.6 (±0.9)
114.8 (±2.4)*	10.8400 (±0.0156)	6.2538 (±0.0089)	5.2005 (±0.0061)	352.5 (±0.8)
119.5 (±2.7)*	10.8188 (±0.0175)	6.2416 (±0.0095)	5.1904 (±0.0055)	350.5 (±0.9)
111.8 (±3.1)*[d]	10.8474 (±0.0217)	6.2581 (±0.0114)	5.2041 (±0.0103)	353.3 (±1.2)
106.1 (±2.5)*[d]	10.8902 (±0.0208)	6.2828 (±0.0098)	5.2246 (±0.0091)	357.5 (±1.1)
96.9 (±2.5)*[d]	10.9351 (±0.0225)	6.3087 (±0.0117)	5.2462 (±0.0089)	361.9 (±1.2)

* NaCl-B2 used as pressure medium.

[d] Decompression.

Table S8. Equation of state parameters of siderite I and II at high pressures and 300 K. V_0: unit cell volume at zero pressure; K_{0T}: isothermal bulk modulus; K_{0T}': pressure derivative of the K_{0T}. In the Birch-Murnaghan EoS fitting, V_0 and K_{0T} have been set as free parameters for both phases. K_{0T}' was set as a free parameter for the siderite I. The EoS parameters for siderite II were derived from having K_{0T}' fixed at 4 or set as a free parameter, respectively. Due to the limited pressure range for the siderite II data, the derived K_{0T}' carries a relatively large uncertainty.

	Siderite I		Siderite II			
	HS	LS	HS	HS*	LS	LS*
V_0 (Å^3)	292.9 (±0.1)	261 (±3)	484 (±9)	489.8 (±2.4)	453 (±8)	459.6 (±3.5)
K_{0T} (GPa)	112 (±2)	131 (±5)	211 (±21)	222 (±13)	234 (±24)	251 (±17)
K_{0T}'	5.2 (±0.2)	5.3 (±0.2)	4.6 (±0.4)	4	4.9 (±0.4)	4

* K_{0T}' fixed at 4.

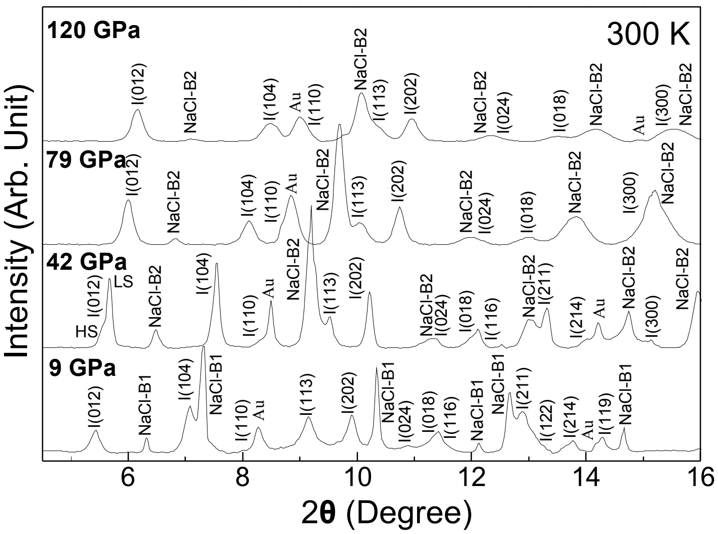

120 GPa
300 K
79 GPa
42 GPa
9 GPa
HS
LS
I(012)
I(104)
I(110)
I(113)
I(202)
I(024)
I(018)
I(116)
I(211)
I(214)
I(300)
I(122)
I(119)
Au
NaCl-B1
NaCl-B2
Intensity (Arb. Unit)
2θ (Degree)
6
8
10
12
14
16

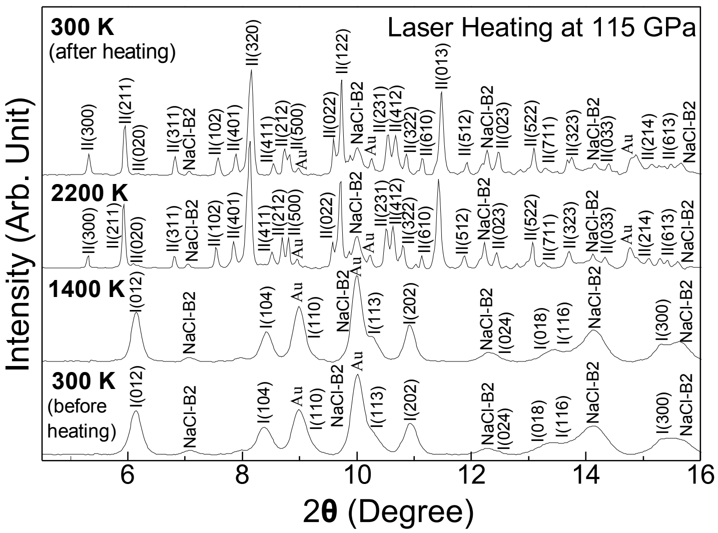

Laser Heating at 115 GPa
300 K
(after heating)
2200 K
1400 K
300 K
(before heating)
Intensity (Arb. Unit)
2θ (Degree)

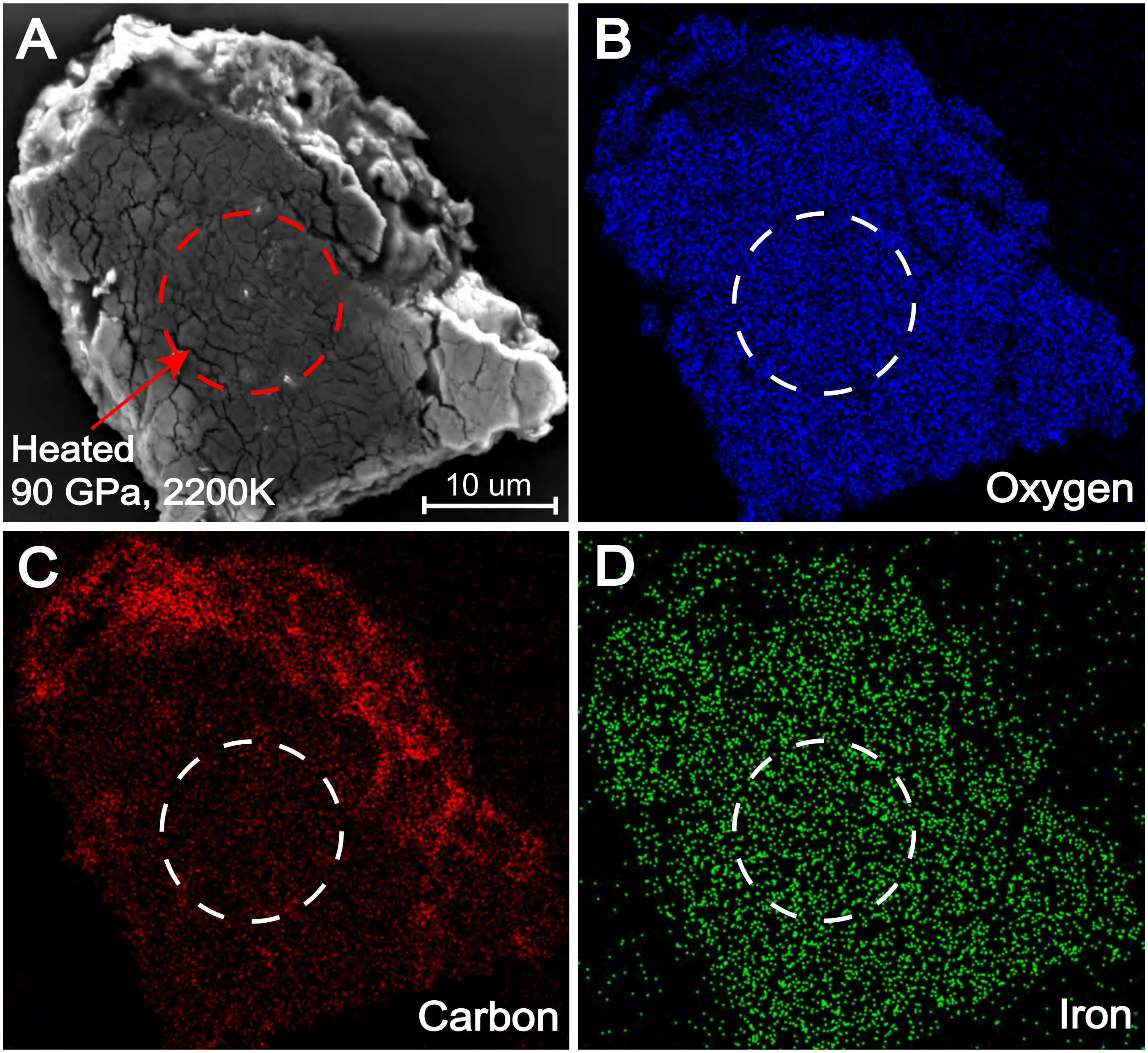
A
Heated
90 GPa, 2200K
10 um
B
Oxygen
C
Carbon
D
Iron
